# GALNT2 targeted by miR-139-5p promotes proliferation of clear cell renal cell carcinoma via inhibition of LATS2 activation

**DOI:** 10.1007/s12672-024-00930-4

**Published:** 2024-03-13

**Authors:** Haisheng Yi, Lingyun Liu, Jingshun Zhang, Kaimin Guo, Yin Cao, Penghao Sun, Hongliang Wang

**Affiliations:** 1 Department of Andrology, The First Hospital of Jilin University, Changchun, 130012 China; 2 Reproductive Medical Center, Department of Obstetrics and Gynecology, The Second Hospital of Jilin University, Changchun, 130041 China

## Abstract

Polypeptide *N*-Acetylgalactosaminyltransferase (GALNTs) are critical enzymes that initiate mucin type-O glycosylation, and are closely associated with the occurrence and development of multiple cancers. However, the significance of GALNT2 in clear cell renal cell carcinoma (ccRCC) progression remains largely undetermined. Based on public multi-omics analysis, GALNT2 was strongly elevated in ccRCC versus adjoining nontumor tissues, and it displayed a relationship with poor overall survival (OS) of ccRCC patients. In addition, GALNT2 over-expression accelerated proliferation of renal cancer cell (RCC) lines. In contrast, GALNT2 knockdown using shRNAs suppressed cell proliferation, and this was rescued by LATS2 knockdown. Similarly, GALNT2 deficiency enhanced p-LATS2/LATS2 expression. LATS2 is activated by phosphorylation (p-LATS2) and, in turn, phosphorylate the downstream substrate protein YAP. Phosphorylated YAP (p-YAP) stimulated its degradation and cytoplasmic retention, as it was unable to translocate to the nucleus. This resulted in reduced cell proliferation. Subsequently, we explored the upstream miRNAs of GALNT2. Using dual luciferase reporter assay, we revealed that miR-139-5p interacted with the 3ʹ UTR of GALNT2. Low miR-139-5p expression was associated with worse ccRCC patient outcome. Based on our experiments, miR-139-5p overexpression inhibited RCC proliferation, and this phenotype was rescued by GALNT2 overexpression. Given these evidences, the miR-139-5p-GALNT2-LATS2 axis is critical for RCC proliferation, and it is an excellent candidate for a new therapeutic target in ccRCC.

**Supplementary Information:**

The online version contains supplementary material available at 10.1007/s12672-024-00930-4.

## Introduction

Renal cell carcinoma (RCC) is a globally widespread malignancy, with the 3rd highest ranking among common urologic cancers. It is only inferior to prostatic and bladder cancers. Clear cell renal cell carcinoma (ccRCC) is the most prevalent histological form of RCC, comprising over 75% of all incidences, and it is the most lethal urological malignancy [1]. Localized ccRCC is typically treated with early surgical resection. However, in case of advanced ccRCC, efficacious treatment is lacking [2]. The median survival of advanced kidney cancer sufferers is less than 12 months and the 5-year overall survival (OS) rate of the same cohort is no higher than 5% [3]. Hence, it is both urgent and critical to examine the mechanisms associated with ccRCC carcinogenesis and progression, as well as explore potential therapeutic targets for improving prognosis of ccRCC patients.

Polypeptide GalNAc Transferase 2 (GALNT2), otherwise known as Polypeptide N-Acetylgalactosaminyltransferase 2 (GalNAc-T2), encodes a member of the glycosyltransferase protein family, which initiates mucin-type *O*-glycosylation of peptides within the Golgi apparatus [4]. Several investigations reported an association between GALNT2 and cancer progression. In colorectal cancer, GALNT2 modified *O*-glycans on AXL was shown to enhance colorectal cancer infiltration [5]. In non-small cell lung cancer, GALNT2 enhances cell proliferation, migration, and infiltration via the *O*-glycosylation of ITGA5 [6]. GALNT2 also facilitates cell proliferation, migration, and infiltration through the *O*-glycosylation and phosphorylation of EGFR in the glioma [7]. However, the precise molecular mechanism underlying the GALNT2-mediated ccRCC progression is not fully elucidated.

The Hippo axis is a highly conserved modulator of cellular growth, proliferation, stem cell function, tissue regeneration, homeostasis, and organ size control [8]. LATS1/2 are critical components of the Hippo axis that inversely modulates the downstream effectors, YAP and TAZ. The Hippo axis dysregulation is strongly associated with multiple cancers [9]. YAP is a transcriptional co-activator, which induces platinum resistance in ovarian tumors [10]. Disturbances in LATS1 and YAP1 expressions are associated with worse ccRCC patient OS [11]. Prior investigations revealed that YAP *O*-GlcNAcylation is essential for high-glucose induced liver tumorigenesis [12]. However, there are no reports of a GALNT2 and LATS2 association.

MicroRNAs (miRNAs) are evolutionarily conserved single-stranded noncoding RNAs that are 18–22 nucleotides in length and negatively modulate gene expression via degradation of target mRNAs or repression of mRNA translation [13]. Owing to the short-binding motif of miRNAs, one miRNA can effectively silence several genes. MicroRNAs heavily contribute to diverse biological phenomena, namely, cell apoptosis, proliferation, differentiation, and immunity [14, 15]. Emerging evidences suggest a strong role of miRNAs in tumorigenesis, cancer progression, and aggressiveness [16]. Thus, herein, we identified potential miRNAs that serve as upstream modulators of GALNT2.

Herein, we detailed a new modulatory mechanism associated with ccRCC progression. We demonstrated that diminished miR-139-5p levels increased GALNT2, which resulted in worse ccRCC patient OS. Furthermore, we revealed that decreased miR-139-5p and/or elevated GALNT2 enhanced RCC proliferation. Our analysis confirmed that the miR-139-5p-GALNT2-LATS2 axis was potentially critical for the ccRCC oncogenesis and progression.

## Methods

### Data accumulation

We obtained mRNA and miRNA sequencing and associated clinical data from the Cancer Genome Atlas (TCGA) and Gene Expression Omnibus (GEO) databases. The UALCAN portal (http://ualcan.path.uab.edu/)-CPTAC (Clinical proteomic tumor analysis consortium) dataset [17] was employed for GALNT2 protein analysis. HPA (https://www.proteinatlas.org/), a freely accessible human proteome database, was employed for analysis of GALNT2 protein expressions and immunohistochemical images from normal and cancerous tissues. We also obtained the single-cell RNA sequencing information of normal renal and ccRCC tissues from HPA and Single cell Portal (https://singlecell.broadinstitute.org/single_cell/study/SCP1288/tumor-and-immune-reprogramming-during-immunotherapy-in-advanced-renal-cell-carcinoma#study-visualize). Subsequently, we performed univariate cox regression analysis of GALNT2 mRNA profile and OS and disease specific survival (DSS) using the “Pathological Stage Plot” module of GEPIA2 [18]. Lastly, the MirDIP: microRNA Data Integration Portal, integrated data from several miRNA-linked databases (BCmicrO|bitargeting_May_2021|CoMeTa|DIANA|MBStar|MirAncesTar|miranda_May_2021|mirbase|miRcode|mirCoX|miRDB_v6|MiRNATIP|mirzag|RNA22|TargetScan_v7_2) was employed for prediction of potential miRNAs that associate with the 3ʹ UTR of GALNT2 [19].

### Cell culture and transfection

The RCC lines (786-O, ACHN, Caki-1 and OS-RC-2) and normal human renal tubular epithelial cell line (HK-2) were acquired from Shanghai Institute of Biochemistry and Cell Biology, Chinese Academy of Sciences, and examined thoroughly for mycoplasma contamination. The nature of cell lines was validated with STR profiling within the last three years. All cells were maintained in RPMI 1640 (Bio-Channel, Nanjing, BC-M-017), MEM (Bio-Channel, Nanjing, BC-M-020), McCoy’s 5A (Bio-Channel, Nanjing, BC-M-041), RPMI 1640 (Bio-Channel, Nanjing, BC-M-017, and DMEM/F12 (Bio-Channel, Nanjing, BC-M-002) with 10% FBS (Bio-Channel, Nanjing, BC-SE-FBS01) and 1% Penicillin–Streptomycin (Genview, GA3502) at 37 °C in 5% CO_2_ humidified chamber. The GALNT2 overexpression lentivirus (oe-GALNT2), vector overexpression lentivirus (oe-vector), short hairpin RNA (shRNA) targeting GALNT2 (shGALNT2), LATS2 (shLATS2), and miR-139-5p mimic were acquired from Gene Pharma (Shanghai, China), then incorporated into various cells for 48 h following kit protocols before further analysis.

### RT-qPCR analysis

Total RNA was isolated from cells using the SanPrep Column microRNA Extraction Kit (B518811, Sangon Biotech, Shanghai, China), and cDNA synthesis was done using Script III First Strand cDNA Synthesis Kit (P118-100, GeneBetter, Beijing). RT-qPCR was conducted with 2 × Sybr Green qPCR Mix (High ROX) (P611-50, GeneBetter, Beijing). Relative gene expression was calculated using the 2 − ΔΔCt formula, and normalized to GAPDH levels. We performed three biological replicates of all samples and controls. The employed primer sequences are described below:

- GALNT2, Forward primers: 5ʹ-GACCCTTACGCCCGCAACAAG-3ʹ,
- Reverse primers: 5ʹ-CCACTGCTTCCGCTGACACTG-3ʹ,
- GAPDH, Forward primers: 5ʹ-GGAGTCCACTGGCGTCTTCA-3ʹ,
- Reverse primers: 5ʹ-GTCATGAGTCCTTCCACGATACC-3ʹ.

### Western blot

Total protein extraction from incorporated cells was conducted using RIPA lysis buffer (WB-0071, Dingguo Changsheng Biotech, Beijing), and protein quantification utilized the BCA protein quantitative kit (BCA01, Dingguo Changsheng Biotech, Beijing). Subsequently, we loaded (20 μg) on 10% SDS-PAGE gel for protein separation, before transfer to PVDF membranes, which were then treated with specific primary antibodies as follows: GALNT2 (Abcam, Shanghai, Ab140637, 1:2000), LATS1 (Abcam, Shanghai, Ab243656, 1:1000), LATS2 (Abcam, Shanghai, Ab243657, 1:1000), p-LATS2 (Proteintech, 28998-1-AP, 1:2000), YAP (Abcam, Shanghai, Ab205270, 1:1000), p-YAP (Abcam, Shanghai, Ab76252, 1:5000), TAZ (Abcam, Shanghai, Ab242313, 1:1000), p-TAZ (Abcam, Shanghai, Ab277791, 1:1000), β-actin (Proteintech, 81115-1-RR, 1:2000), and GAPDH (PROTEINTECH; 60004-1, 1:5000). Protein visualization utilized the ECL reagent (ECL-0111, Dingguo Changsheng Biotech, Beijing) and protein quantification was performed using Image J, with GAPDH or β-actin as the control.

### MTT cell proliferation and cytotoxicity assay

Cell survivability was assessed via the MTT Cell Proliferation and Cytotoxicity Assay Kit (Solarbio, M1020). Approximately 3000 cells/well were seeded in 96-well plates, and incubated at 37 °C in a 5% CO_2_ incubator for approximately 24, 48, 72, and 96 h prior to treatment with specified compounds. Following treatment, 20 μl MTT solution was introduced to the cells, followed by a 4 h incubation at 37 °C in a 5% CO_2_ incubator. The reaction was terminated by removing the media, then 150 μl dimethylsulfoxide (DMSO) was introduced and optical density was determined using Spectramax (Molecular Devices) at 490 nm. All samples were assayed thrice.

### Colony formation assays

Cells were maintained on 6-well plates (200 cells per well), with fresh medium every 3rd day for 14 days. Next, they underwent a 15 min fixation in 4% paraformaldehyde (PFA), and staining with crystal violet. They were then imaged and quantified.

### Brdu incorporation assay

We acquired the anti-Brdu (Ab6326) and rhodamine anti-mouse antibodies (SA00007-1) from Abcam and proteintech, respectively. Following a 16–18 h incubation in 10 μM BrdU, the cells underwent fixation in 4% PFA, before BrdU incorporation assessment with an immunofluorescence microscope.

### Cell apoptosis evaluation

Cell apoptosis was assessed using flow cytometry (FC) via the Annexin V-FITC Apoptosis Detection Kit (Beyotime, C1062L). In short, cells were maintained for 24 h in 96-well plates, before introduction of 5 μL Annexin V-FITC and 10 μL propidium iodide (PI), and incubation for 15 min at 37 °C. We next assessed the amount of early (Annexin V-FITC(+)/PI(−)) and late (Annexin V-FITC(+)/PI(+)) apoptotic cells using FC.

### Luciferase assay

The dual-luciferase vectors were acquired from Jiangsu Dongxuan Gene Technology Co. Ltd., and harbored the 3ʹ-UTR of GALNT2 with seed sequences. The corresponding mutant vectors were generated by ligating the 3-bp mutations with the aforementioned seed sequences. Next, the GALNT2/mutant vectors and miR-139-5p mimics were co-incorporated into Caki-1 and OS-RC-2 cell lines by Gene Pharma (Gene Pharma; Shanghai GenePharma Co. Ltd., CHINA). Luciferase activity quantification was performed 48 h later with the use of Dual-Luciferase Reporter Assay System (Molecule Device, Molecular Devices, LLC., U.S.A.).

### Statistical analysis

All experiments were performed in three biological replicates. Data were analyzed using SPSS 16.0, and are provided as mean ± SD. Inter-group assessments utilized the unpaired Student’s t-test. GALNT2 content and clinicopathological features were assessed using the χ^2^ test. Survival curves were plotted and assessed following the Kaplan–Meier technique and the log-rank test, respectively. Association between GALNT2 and miR-139-5p contents was assessed via the Spearman’s correlation coefficient. ns p > 0.05. was not significant. *p < 0.05, **p < 0.01, ***p < 0.001 were set as the significance threshold.

## Results

### High GALNT2 expression level linked to poor prognosis of ccRCC

**Fig. 1:**
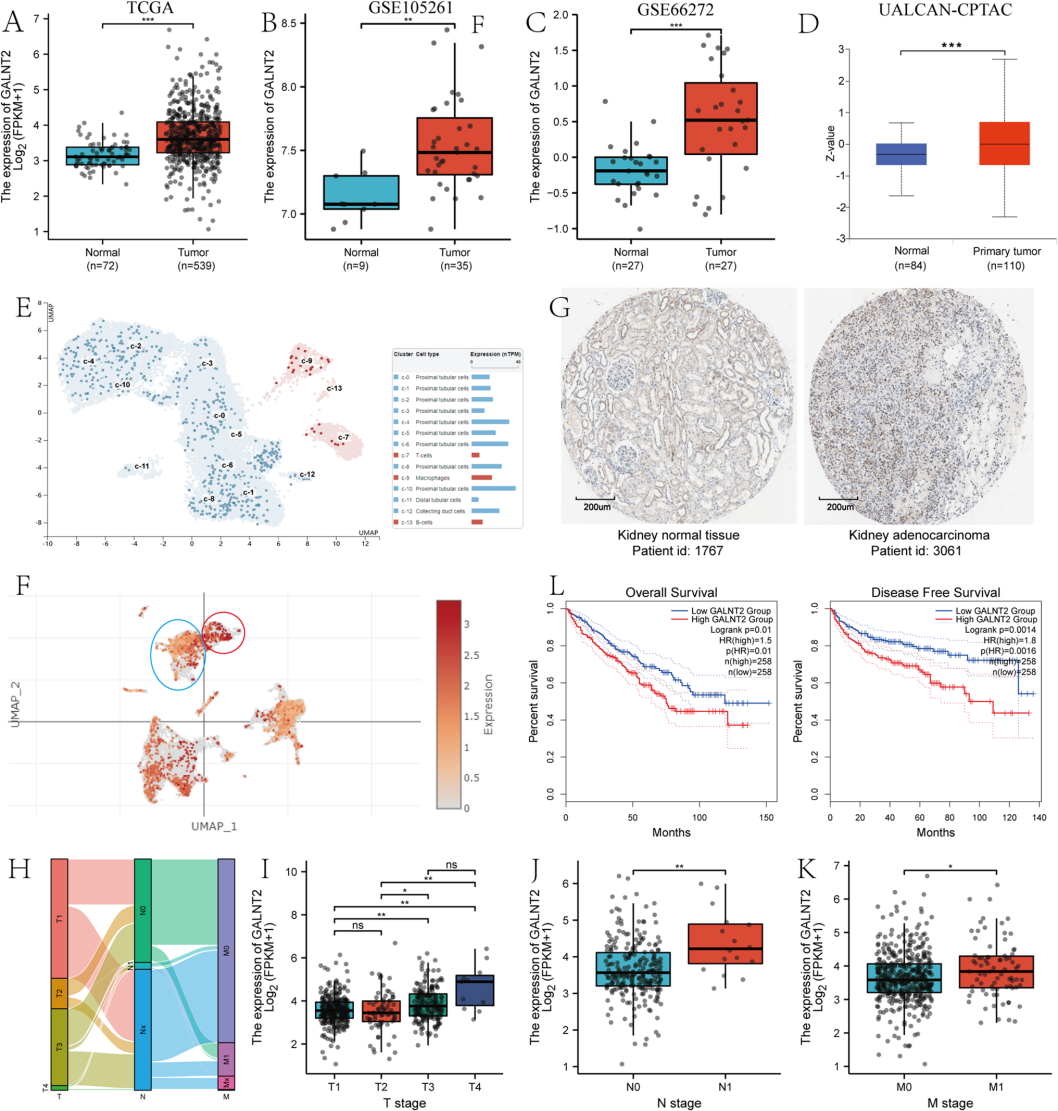
GALNT2 was elevated in ccRCC tissues, and was correlated with worse patient outcome. **A**–**C** GALNT2 mRNA levels in tumor and adjoining normal tissues (NTs) in TCGA, GSE105261, and GSE66276. **D** GALNT2 protein expressions in tumor and NTs in UALCAN. **E** The single cell GALNT2 content in kidney in HPA. **F** The single cell GALNT2 content in varying ccRCC stages in Single cell Portal. Red circle indicated single cell of late stage ccRCC; blue circle indicated single cell of early stage ccRCC. **G** GALNT2 protein expression in tumor and adjoining normal tissues in HPA, as evidenced by immunohistochemistry. **H** The Sankey diagram display of the proportions of TNM stages in TCGA. **I**–**K** The GALNT2 expression in TNM stages in TCGA. **L** Elevated GALNT2 expression correlates with poor overall survival (OS) and disease-free survival (DFS) in GEPIA, as evidenced by univariate analysis

We firstly employed the TCGA and GEO database for GALNT2 gene expression analysis in ccRCC tumor and adjoining normal tissue (NT). Relative to NT, GALNT2 contents were markedly enhanced in tumor samples, as depicted in Fig. 1A–C. Subsequently, we verified the enhanced GALNT2 protein levels in tumor versus NT samples in UALCAN (Fig. 1D). Using single-cell sequencing, we next revealed that GALNT2 was primarily present on the proximal tubular of the kidney in HPA (Fig. 1E, https://www.proteinatlas.org/ENSG00000143641-GALNT2/single+cell+type/kidney). Moreover, GALNT2 in the early stage was lower than the later ccRCC stage (Fig. 1F, Additional file 3: Fig. 1). Similarly, based on IHC results, GALNT2 was substantially elevated in tumor versus NT in the HPA website (Fig. 1G, https://www.proteinatlas.org/ENSG00000143641-GALNT2/tissue/kidney#img, https://www.proteinatlas.org/ENSG00000143641-GALNT2/pathology/renal+cancer#img). Sankey diagram showed a correspondence to TNM stage of ccRCC in TCGA (Fig. 1H). Furthermore, as the ccRCC TNM stage advanced, the GALNT2 content was enhanced (Fig. 1I–K). Table 1 illustrates patient demographics from TCGA. We next conducted OS and disease-free survival (DFS) analyses based on the GALNT2 expression (0%–50% vs 50%–100%) using GEPIA2. Our Cox regression analysis revealed that enhanced GALNT2 levels were strongly associated with worse OS (HR = 1.5, p = 0.01) and DFS (HR = 1.8, p = 0.0016) in ccRCC patients (Fig. 1L). Collectively, these results indicated that GALNT2 maybe modulate ccRCC progression.

**Table 1:** Correlation between GALNT2 content and clinicopathological features of ccRCC patients

Characteristic	Levels	Low expression of GALNT2^a^	High expression of GALNT2^a^	p
n		269	270	
Gender, n (%)	Female	110 (20.4%)	76 (14.1%)	0.003
	Male	159 (29.5%)	194 (36%)	
Race, n (%)	Asian	3 (0.6%)	5 (0.9%)	0.692
	Black or African American	30 (5.6%)	27 (5.1%)	
	White	230 (43.2%)	237 (44.5%)	
Age, n (%)	≤ 60	137 (25.4%)	132 (24.5%)	0.698
	> 60	132 (24.5%)	138 (25.6%)	
Age, mean ± SD		60.58 ± 11.89	60.67 ± 12.32	0.928
T stage, n (%)	T1	152 (28.2%)	126 (23.4%)	0.002
	T2	42 (7.8%)	29 (5.4%)	
	T3	73 (13.5%)	106 (19.7%)	
	T4	2 (0.4%)	9 (1.7%)	
N stage, n (%)	N0	131 (51%)	110 (42.8%)	0.012
	N1	3 (1.2%)	13 (5.1%)	
M stage, n (%)	M0	220 (43.5%)	208 (41.1%)	0.118
	M1	32 (6.3%)	46 (9.1%)	
Pathologic stage, n (%)	Stage I	149 (27.8%)	123 (22.9%)	0.015
	Stage II	35 (6.5%)	24 (4.5%)	
	Stage III	51 (9.5%)	72 (13.4%)	
	Stage IV	34 (6.3%)	48 (9%)	
OS event, n (%)	Alive	198 (36.7%)	168 (31.2%)	0.006
	Dead	71 (13.2%)	102 (18.9%)	
DSS event, n (%)	Alive	226 (42.8%)	194 (36.7%)	< 0.001
	Dead	36 (6.8%)	72 (13.6%)	
PFI event, n (%)	Alive	210 (39%)	168 (31.2%)	< 0.001
	Dead	59 (10.9%)	102 (18.9%)	

^a^The division of high-expression and low-expression of GALNT2 was based on the median cutoff value 50%

### GALNT2 overexpression augmented RCC cell proliferation

**Fig. 2:**
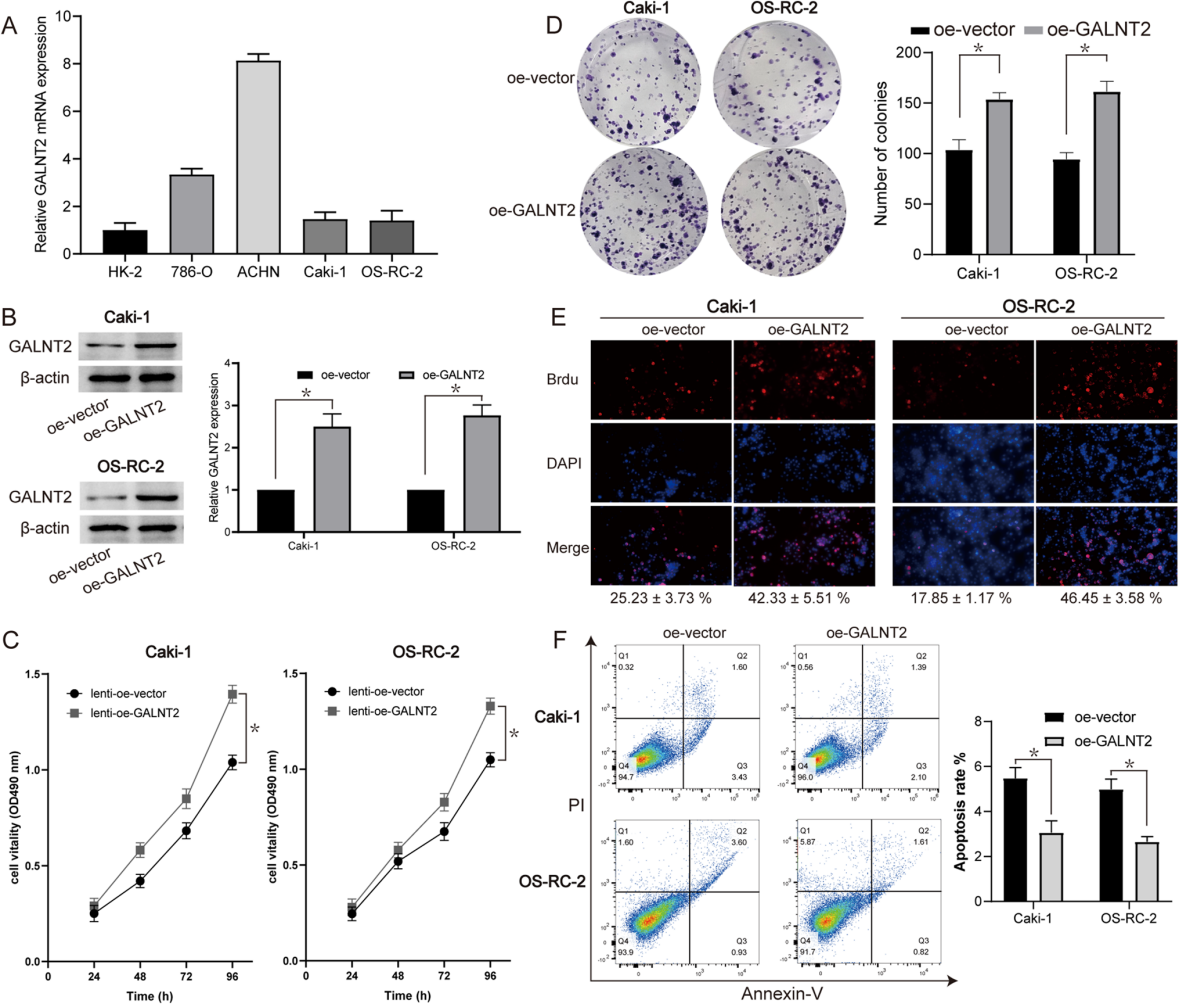
GALNT2 overexpression induced RCC cell proliferation in vitro. **A** GALNT2 expression analysis in RCC cell lines via RT-qPCR. **B** GALNT2 expression analysis in RCC cell lines via Western blot. **C** MTT cell proliferation assay. **D** Colony formation assay. **E** Brdu cell proliferation assay. **F** Flow cytometric analysis of cell apoptosis

To further elucidate the physiological mechanisms behind the GALNT2-mediated acceleration of ccRCC progression, we examined the GALNT2 transcript levels in RCC cell lines and renal proximal tubular epithelial cell using RT-qPCR (Fig. 2A). We overexpressed GALNT2 in different RCC lines in vitro (Fig. 2B). Then, using MTT, colony formation, and Brdu assays, we confirmed that GALNT2 overexpression significantly enhanced RCC proliferation (Fig. 2C–E). In the meantime, using flow cytometry, we revealed that GALNT2 overexpression strongly diminished cell apoptosis (Fig. 2F). Taken together, GALNT2 overexpression markedly enhanced RCC cell proliferation.

### GALNT2 modulated LATS2 activation in ccRCC

**Fig. 3:**
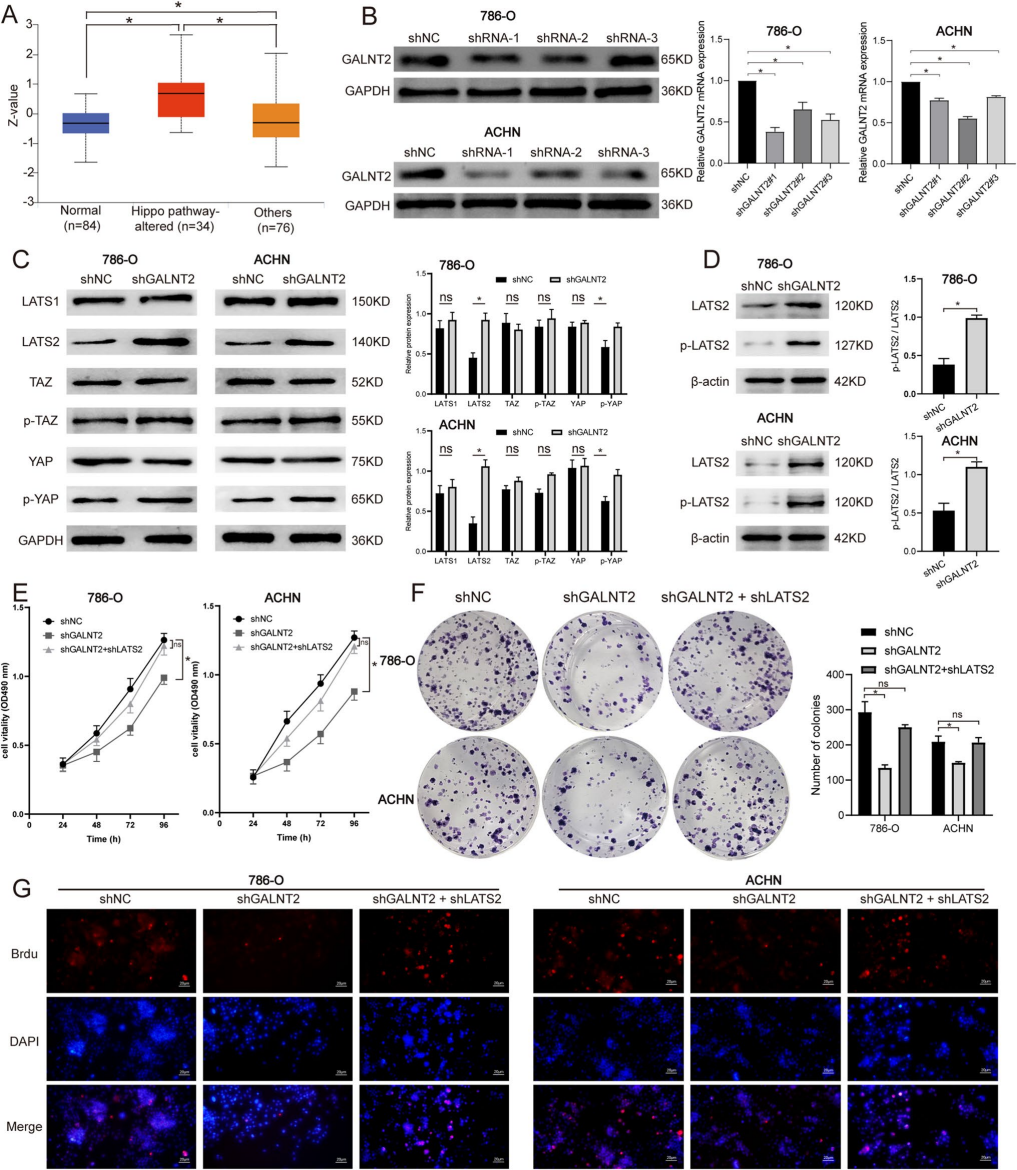
GALNT2 knockdown inhibited cell proliferation, and LATS2 overexpression rescued the proliferative properties of RCC cells. **A** GALNT2 protein expression profile, based on the Hippo pathway status in UALCAN. **B** GALNT2 expression analysis in RCC cell lines, as evidenced by Western blot. **C** Effects of GALNT2 knockdown on the expression of Hippo pathway proteins. **D** Effects of GALNT2 knockdown on the expression of p-LATS2/LATS2. **E** MTT cell proliferation assay. **F** Colony formation assay. **G** Brdu cell proliferation assay

To explore the mechanism behind the GALNT2-mediated regulation of cell proliferation, we examined the downstream signaling pathways. We acquired the GALNT2 protein expression data, in correlation with multiple signaling network, from the UALCAN database. The Hippo pathway showed the most significant p-value, and was therefore chosen for further analysis. The GALNT2 proteomic expression profile in UALCAN indicated that dysregulation of the Hippo pathway was related to GALNT2 levels in ccRCC (Fig. 3A). Next, we knocked down GALNT2 in RCC lines (Fig. 3B), and assessed Hippo pathway-relevant key protein expressions. We revealed that the LATS2 and p-YAP proteins were strongly enhanced in the GALNT2-knockdown group (Fig. 3C). In addition, phosphorylated LATS2 (p-LATS2) is the active form, and the p-LATS2 protein was markedly enhanced in the GALNT2-knockdown group (Fig. 3D). Next, using MTT, colony formation, and Brdu assays, we revealed that the GALNT2 knockdown severely suppressed cell proliferation. However, LATS2 knockdown rescued this phenotype (Fig. 3E–G). Collectively, these evidences suggested that GALNT2 reduced LATS2 activation to enhance RCC proliferation.

### miR-139-5p was an upstream modulator of GALNT2 in ccRCC

**Fig. 4:**
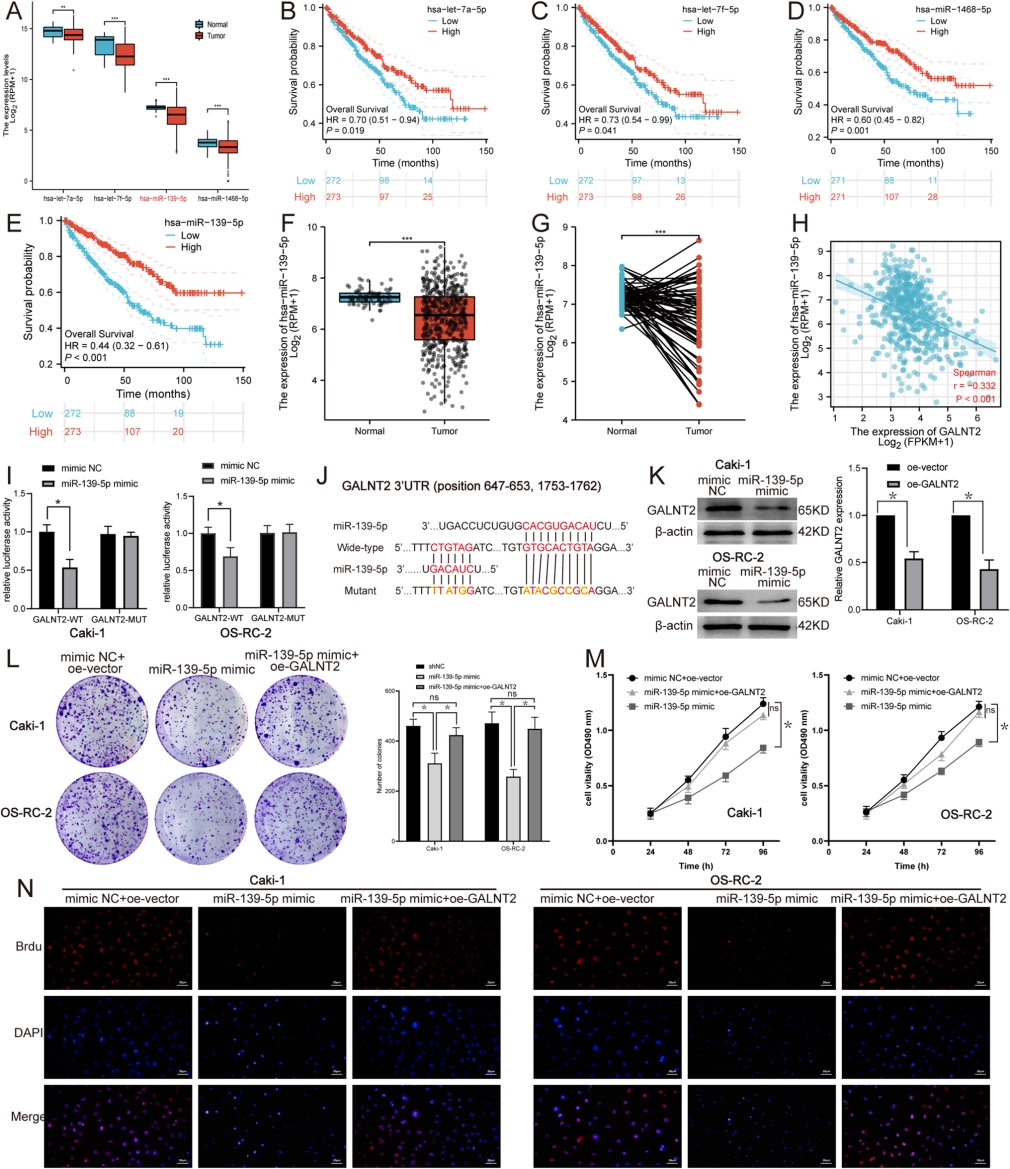
miR-139-5p negatively regulated cell proliferation, and this can be rescued via GALNT2 overexpression. **A** Expression of 4 candidate miRNAs in TCGA. **B**–**E** 4 candidate miRNAs whose elevated levels indicated enhanced overall survival (OS) of ccRCC patients in TCGA. **F**, **G** miR-139-5p expression in unpaired and paired ccRCC samples in TCGA. **H** Expression correlation of GALNT2 and miR-139-5p in ccRCC in TCGA. **I** Luciferase reporter assay. **J** A schematic representation of the putative binding site of miR-139-5p in the GALNT2 3ʹ-UTR. **K** The expression levels of GALNT2 after miR-139-5p mimic transfection. **L** Colony formation assay. **M** MTT cell proliferation assay. **N** Brdu cell proliferation assay

Accumulating evidence implicated that miRNA negatively regulated gene expression. Herein, we predicted the upstream miRNAs of GALNT2. After searching the mirDIP 4.1 with the keywords “GALNT2” and “Top 5% Score class”, it was found that 296 miRNAs targeted to GALNT2 (Additional file 1). Enhanced GALNT2 expression was correlated with worse ccRCC patient outcome. So, we filtered out 4 out of the 296 miRNAs in mirDIP 4.1, which high expression indicated enhanced OS of ccRCC patients (Table 2, Fig. 4A–E). We finally selected miR-139-5p for further exploration based on the published literature. Figure 4F, G depicted markedly enhanced miR-139-5p contents in unpaired and paired ccRCC samples in TCGA (Fig. 4F, G). Spearman correlation analysis demonstrated that GALNT2 and miR-139-5p expressions in ccRCC were inversely proportional in TCGA (Fig. 4H). Similarly, dual luciferase reporter assay revealed that miR-139-5p diminished GALNT2 levels in RCCs (Fig. 4I). Within the 3ʹ-UTR of GALNT2, we identified two potential miR-139-5p-binding sites (Fig. 4J). MiR-139-5p overexpression in RCCs diminished GALNT2 protein expression (Fig. 4K). Lastly, using MTT, colony formation, and Brdu assays, we revealed that miR-139-5p overexpression strongly suppressed cell proliferation, whereas, GALNT2 overexpression rescued this phenotype (Fig. 4L–N). In conclusion, herein, we identified miR-139-5p as an upstream negative factor of GALNT2 in RCCs.

**Table 2:** The 4 candidate miRNAs that potentially targeted GALNT2 in ccRCC, as evidenced by *mirDIP 4.1*

MicroRNA	Integrated Score	Number of Sources	Score Class	Sources
hsa-let-7a-5p	0.706060	12	High	CoMeTa|Cupid|DIANA|MBStar|MirAncesTar|miRDB_v6|MiRNATIP|miRTar2GO| mirzag|MultiMiTar|RNA22|TargetScan_v7_2
hsa-let-7f-5p	0.686622	12	High	BCmicrO|CoMeTa|DIANA|MBStar|MirAncesTar|miRDB_v6|MiRNATIP|miRTar2GO |mirzag|MultiMiTar|RNA22|TargetScan_v7_2
hsa-miR-139-5p	0.430561	10	High	BCmicrO|bitargeting_May_2021|MirAncesTar|miranda_May_2021|mirbase|miRcode|mirCoX|MirSNPInTarget|miRTar2GO|PITA_May_2021
hsa-miR-1468-5p	0.159518	6	High	bitargeting_May_2021|MirAncesTar|miranda_May_2021|mirCoX|MiRNATIP|RNA22

## Discussion

Glycosylation was a widespread modification in various cellular and biological processes. GALNT2 belonged to the GalNAc-type *O*-glycosylation gene family, and modulated multiple forms of cancers. Glycosyltransferases were excellent prognostic bioindicators of pan-cancer patient OS [20]. The contents of varying GalNAc-transferases were potentially relevant to the progression and poor outcome of epithelial ovarian cancer patients [21]. GALNT2 modified O-glycosylation and EGFR activity enhanced oral squamous cell carcinoma migration and invasion [22]. GALNT2 also activated the Notch/Hes1-PTEN-PI3K/Akt axis to induce cell proliferation, migration, and invasion in lung adenocarcinoma [23]. Despite these reports, the mechanism underlying GALNT2 action remained unclear in ccRCC progression. Herein, we demonstrated that GALNT2 was ubiquitous among ccRCC tissues, and strongly associated with poor ccRCC patient outcome. We next examined the significance of GALNT2 in ccRCC progression. Elevated GALNT2 levels accelerated RCC proliferation in a series of in vitro experiments. Then elevated GALNT2 levels stimulated the Hippo pathway to enhance RCC proliferation. GALNT2 knockdown marked enhanced LATS2, p-LATS2 and p-YAP levels. Moreover, GALNT2 knockdown strongly suppressed cell proliferation, and this was rescued by LATS2 knockdown. Our analysis identified LATS2 as a key molecule that was targeted by GALNT2 in ccRCC progression. Unfortunately, owing to limitations in experimental condition, we failed to identify the direct interaction sites between GALNT2 and LATS2. However, we have several possible hypotheses. LATS2 may be glycosylated by GALNT2, which, in turn, may inhibit degradation or induce activation of LATS2. However, one study revealed that *O*-GlcNAcylation of LATS2 by OGT (O-Linked *N*-Acetylglucosamine (GlcNAc) Transferase) strongly inhibits its activity [24]. GALNT2 (GalNAc-Transferase) and *O*-GlcNAcylation (GlcNAc Transferase) may ecounter competitive inhibition. Alternately, the Hippo pathway-related upstream proteins may be glycosylated and activated by GALNT2, which may result in LATS2 activation. LATS1/2 and YAP/TAZ are key molecules in ccRCC progression. Prior investigation revealed that LATS1/2 and active YAP dysregulation are sufficient to initiate renal tumor formation in mice [25]. Overall, LATS2 is a potentially promising target of GALNT2 in ccRCC progression.

**Fig. 5:**
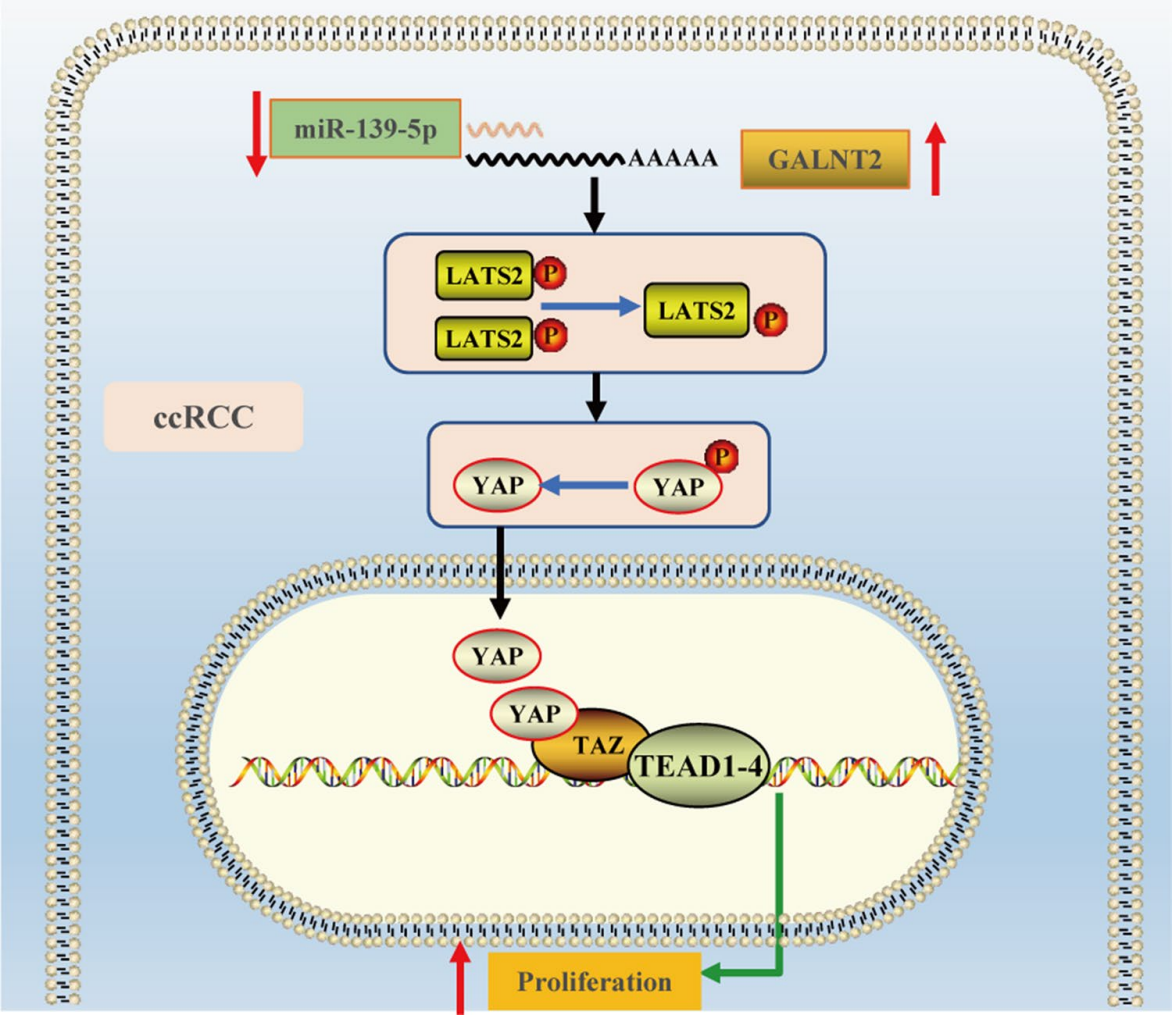
A schematic diagram of the the miR-139-5p-GALNT2-LATS2 axis-based mechanism regulating ccRCC progression

MiR-139-5p exhibited scarce expression in ccRCC tissues, and indicated a poor ccRCC prognosis. Based on our dual luciferase reporter assay analysis, miR-139-5p interacted with the 3ʹ UTR of GALNT2. Hence, it was identified as an upstream modulatory factor of GALNT2. MiR-139-5p is a tumor suppressor, and its expression is strongly diminished in the vast majority of human tumors [26]. MiR-139-5p targeted GABRA1 to suppress glioma cell proliferation [27]. Exosomal miR-139-5p suppressed bladder cancer malignancy by targeting PRC1 [28]. MiR-139-5p was also a tumor suppressor, which targets TOP2A and PXN in RCC [29, 30]. Herein, we presented GALNT2 as a new target of miR-139-5p, thereby interpreting a potential mechanism of cell proliferation in ccRCC. Based on this, we reasoned that the miR-139-5p-GALNT2-LATS2 axis accelerated cell proliferation in ccRCC (Fig. 5). Based on our findings, the 3ʹ-UTR of several Hippo pathway-related proteins, namely, STK3, STK4, LATS1, and YAP1, potentially bind miR-139-5p (Additional file 2). In our future investigation, we plan to further explore the modulatory role between miR-139-5p and the Hippo pathway.

In this paper, OS and DSS indicators were used to evaluate the impact of GALNT2 on ccRCC prognosis. However, disease specific survival (DSS) was not collected by the TCGA study. The source of DSS came from a publication [31] that estimated DSS data, and the authors of that study cautioned on the utilization of this data point, depending on the specific tumor type. Therefore, the DSS was estimated and that this was a limitation of this study.

Tumor cells were controlled by multiple regulatory mechanisms during initiation and progression. In this study, we found that GALNT2 acted as a key regulator for the initiation and progression of ccRCC. In further mechanistic studies, we found that the disorder of the miR-139-5p-GALNT2-LATS2 regulatory axis were widespread in ccRCC and play important roles in the cell proliferation of ccRCC. Our study provided insight into new cancer therapeutic strategies for ccRCC targeting the miR-139-5p-GALNT2-LATS2 axis.

## Conclusions

GALNT2 was highly expressed in tumor versus NT samples. Hence, GALNT2 may be a robust stand-alone indicator of worse ccRCC patient OS. Using functional and molecular experiments, we revealed that GALNT2 accelerated RCC proliferation in vitro, as well as tumor growth in vivo. We also identified LATS2 as a key downstream signaling molecule of GALNT2, and miR-139-5p as an upstream modulator of GALNT2 in ccRCC. More importantly, herein, we demonstrated for the first time that the miR-139-5p-GALNT2-LATS2 axis accelerated ccRCC progression. Hence, the miR-139-5p-GALNT2-LATS2 axis may be a potential target for future ccRCC therapy.

### Supplementary Information

**Additional file 1.** 296 miRNAs targeted to GALNT2 in mirDIP.

**Additional file 2.** Potential proteins in hippo bind to miR-139-5p in mirDIP.

**Additional file 3.**Supplement figure 1, Gels and blots and GALNT2 mutant sequence.

## Data Availability

All data generated or analysed during this study are included in the article/additional material. All referenced public databases can be found in the article. Further inquiries can be directed to the corresponding authors.
