# Supplementary material for: GALNT2 targeted by miR-139-5p promotes proliferation of clear cell renal cell carcinoma via inhibition of LATS2 activation
Source: Discov Oncol. 2024 Mar 13;15:73. doi: 10.1007/s12672-024-00930-4 (PMC10937861; doi:10.1007/s12672-024-00930-4)
Supplement: Supplementary file 3 — Additional file 3.Supplement figure 1, Gels and blots and GALNT2 mutant sequence. [file 12672_2024_930_MOESM3_ESM.pdf]

Supplementary Figure 1

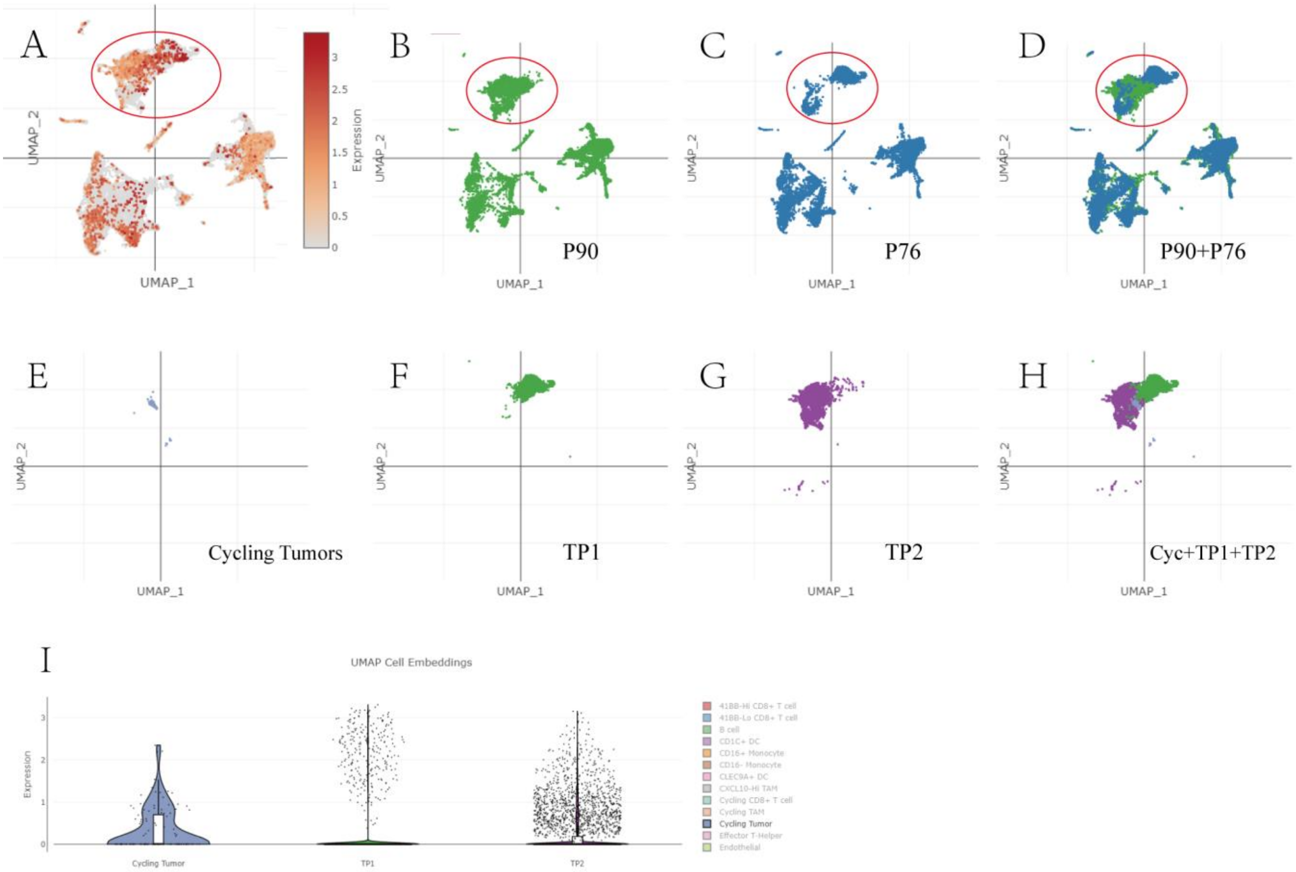

Supplementary Fig.1. The single cell expression levels of GALNT2 in different ccRCC stage in Single cell Portal. (https://singlecell.broadinstitute.org/single_cell/study/SCP1288/tumor-and-immune-reprogramming-during-immunotherapy-in-advanced-renal-cell-carcinoma#study-visualize)

Sample : P90, Study ID: 0600890_T1 , Age: 71, Sex: Female, Biopsy site: Kidney, Histology: ccRCC, Grade: 4, Stage: I, Cells: 8426.

Sample : P76, Study ID:0600876_T1 , Age: 68, Sex: Female, Biopsy site: Kidney, Histology: ccRCC, Grade: 4, Stage: IV, Cells: 7912.

(A) The UMAP of the expression of GALNT2, (B) The UMAP of P90, (C) The UMAP of P76, (D) The UMAP of P90+P96, (E) The UMAP of the cycling tumor cells, (F) The UMAP of TP1, (G) The UMAP of TP2, (H) The UMAP of cycling tumor cells, TP1 and TP2, (I) The GALNT2 expression of cycling tumor cells, TP1 and TP2.

GALNT2 targeted by miR-139-5p promotes proliferation of clear cell renal cell carcinoma via inhibition of LATS2 activation

Gels and Blots

WB Marker

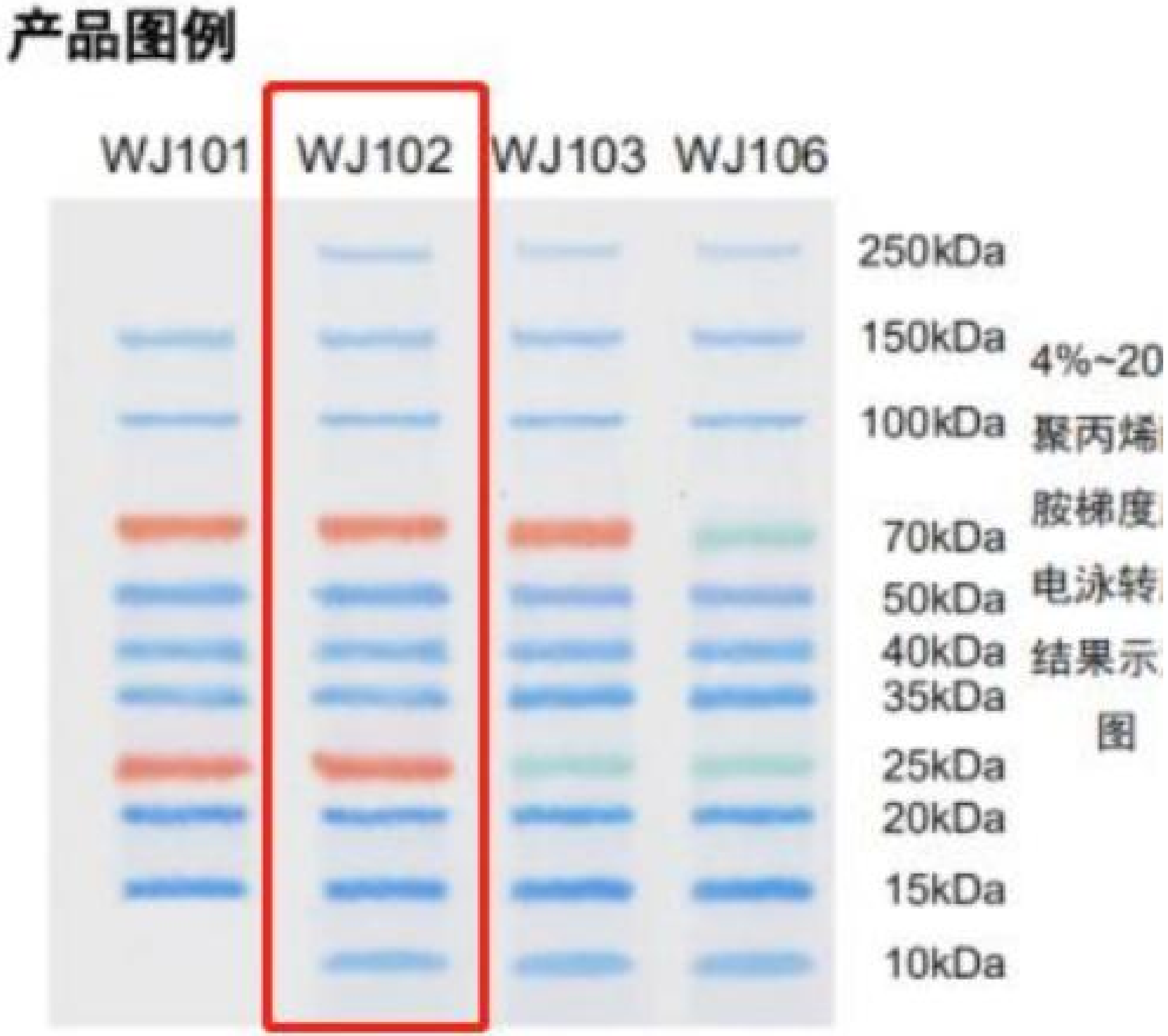
产品图例
WJ101
WJ102
WJ103
WJ106
250kDa
150kDa
100kDa
70kDa
50kDa
40kDa
35kDa
25kDa
20kDa
15kDa
10kDa
4%~20
聚丙烯
胺梯度
电泳转
结果示
图

Figure 2B

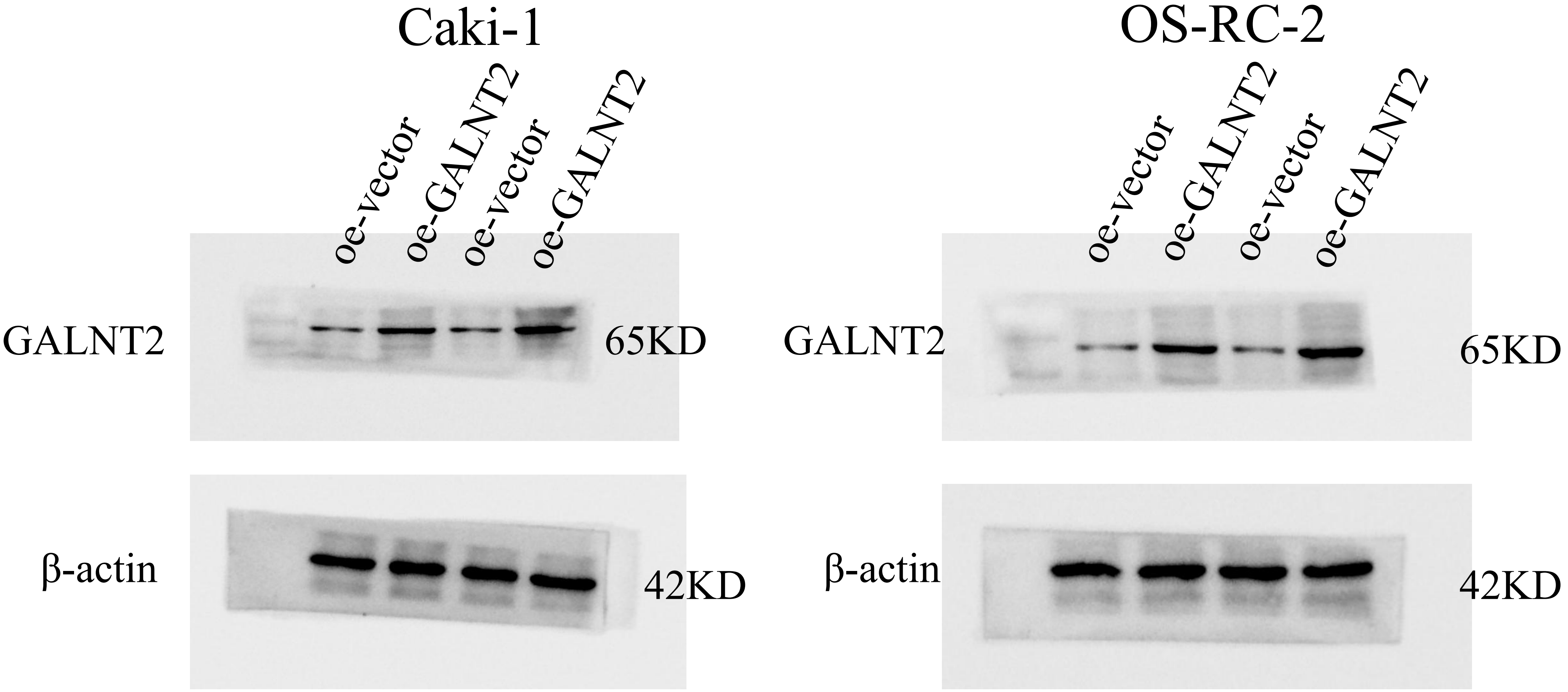
Caki-1
OS-RC-2
oe-vector
oe-GALNT2
oe-vector
oe-GALNT2
oe-vector
oe-GALNT2
oe-vector
oe-GALNT2
GALNT2
65KD
GALNT2
65KD
β-actin
42KD
β-actin
42KD

Figure 3B

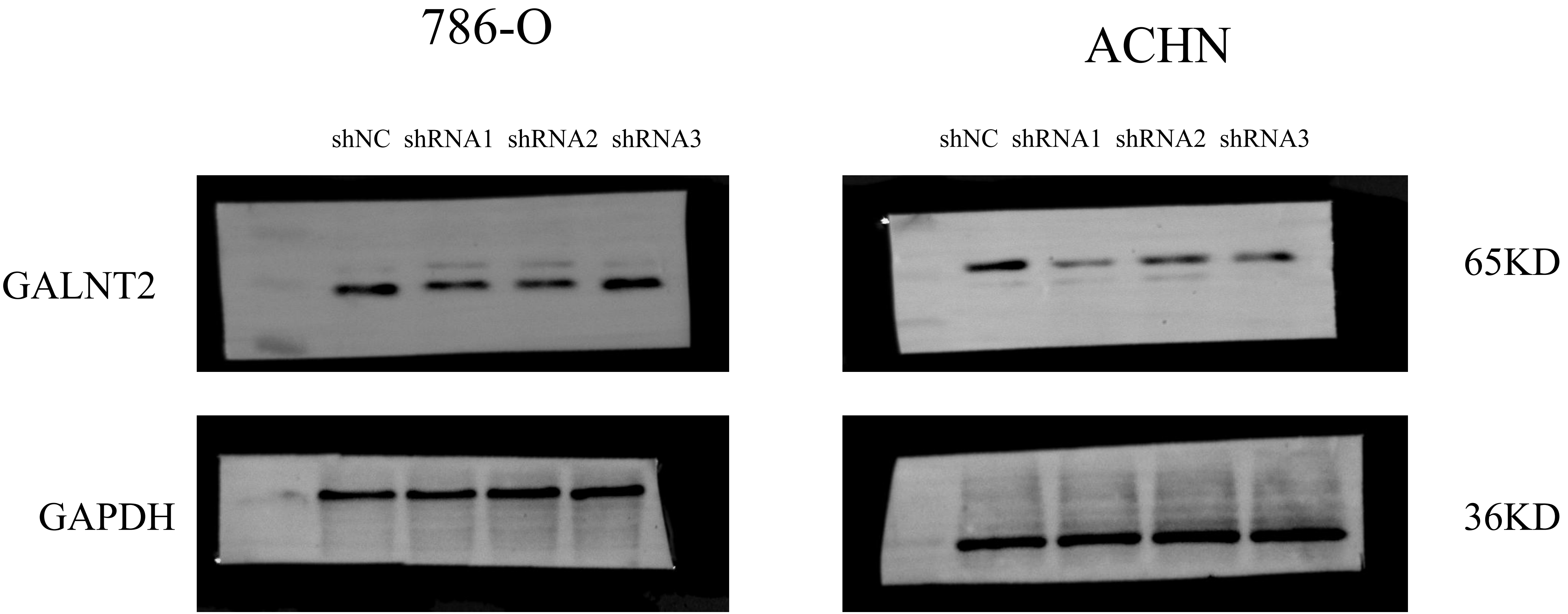

786-O
ACHN
shNC shRNA1 shRNA2 shRNA3
shNC shRNA1 shRNA2 shRNA3
GALNT2
GAPDH
65KD
36KD

Figure 3C

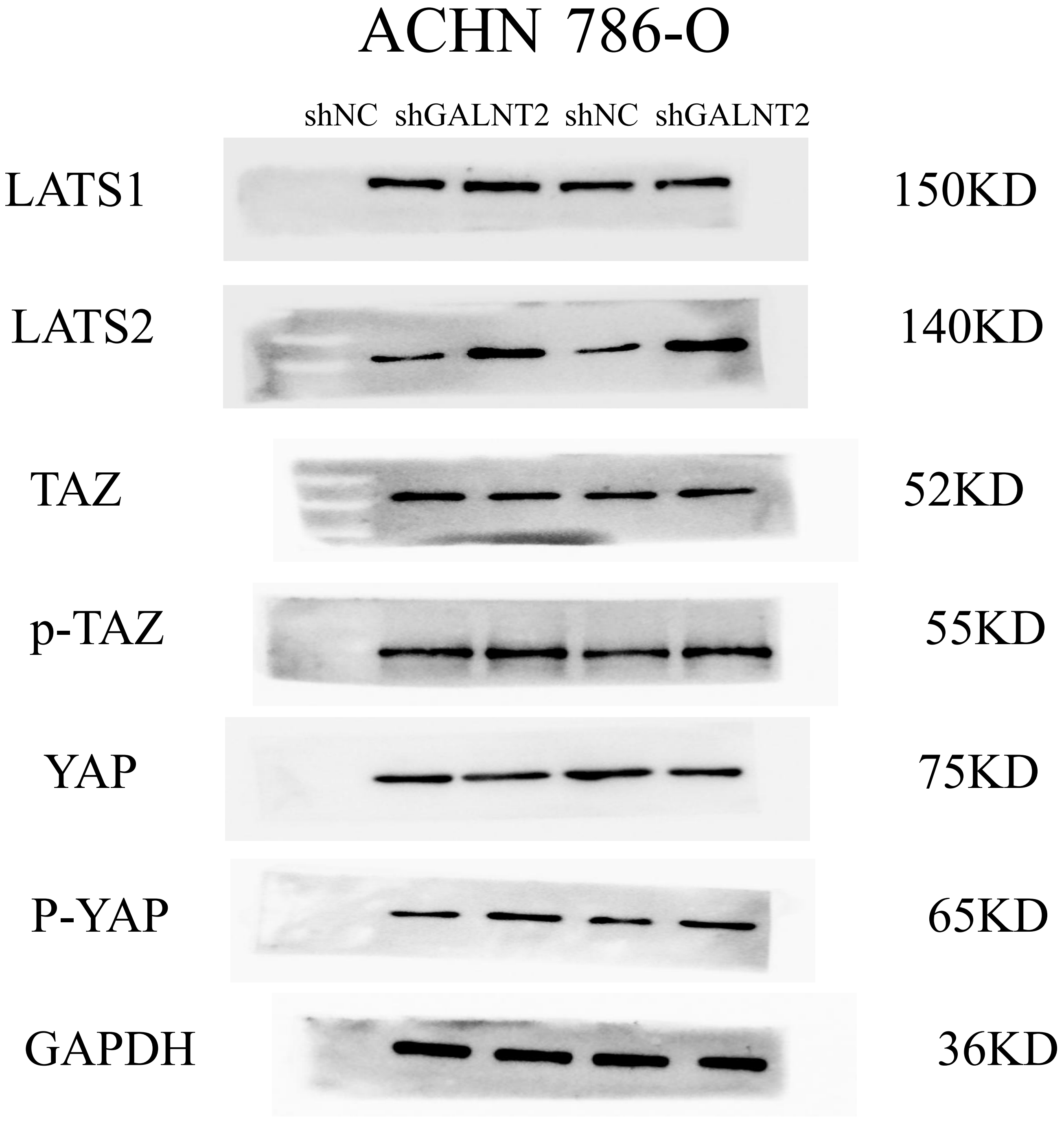

ACHN 786-O
shNC shGALNT2 shNC shGALNT2
LATS1
150KD
LATS2
140KD
TAZ
52KD
p-TAZ
55KD
YAP
75KD
P-YAP
65KD
GAPDH
36KD

Figure 3D

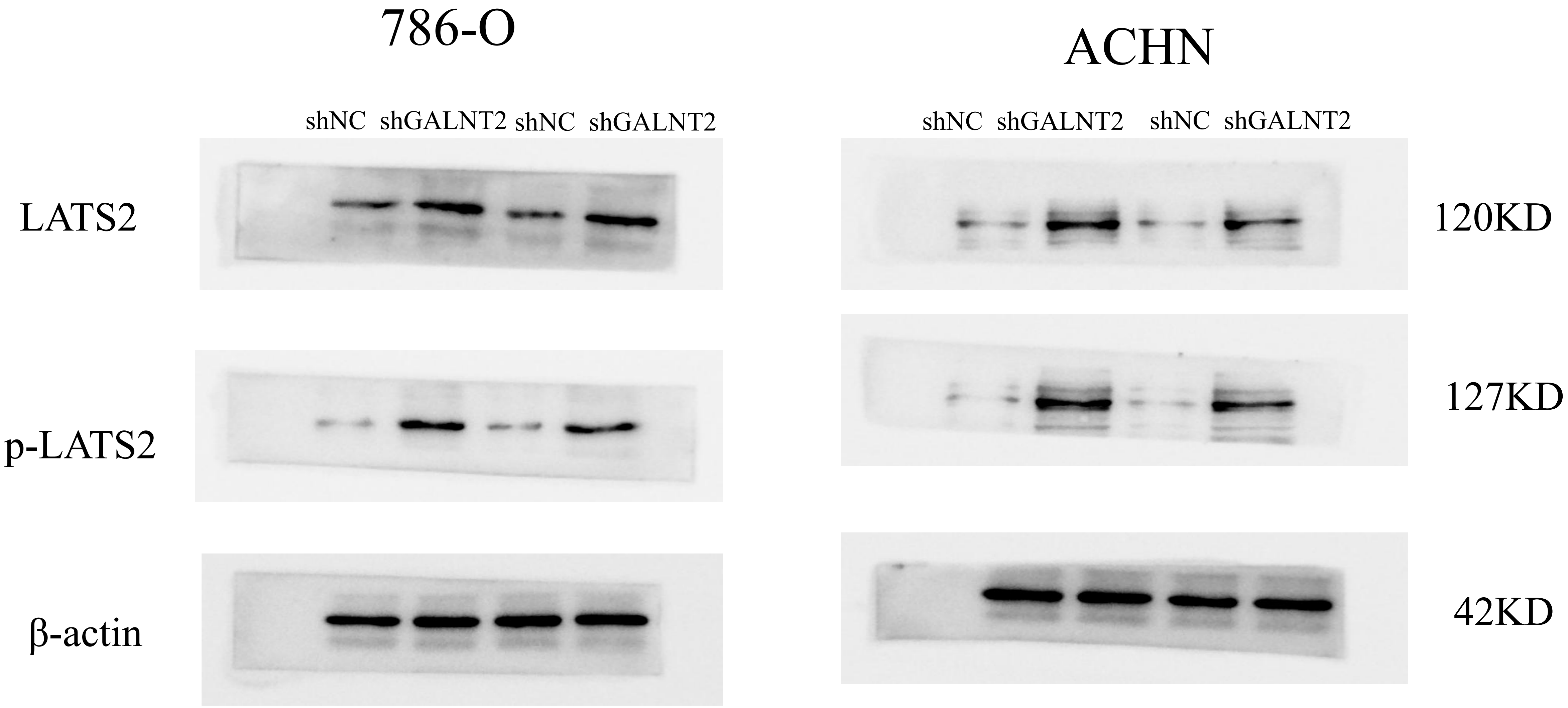
786-O
ACHN
shNC shGALNT2 shNC shGALNT2
shNC shGALNT2 shNC shGALNT2
LATS2
p-LATS2
β-actin
120KD
127KD
42KD

Figure 4K

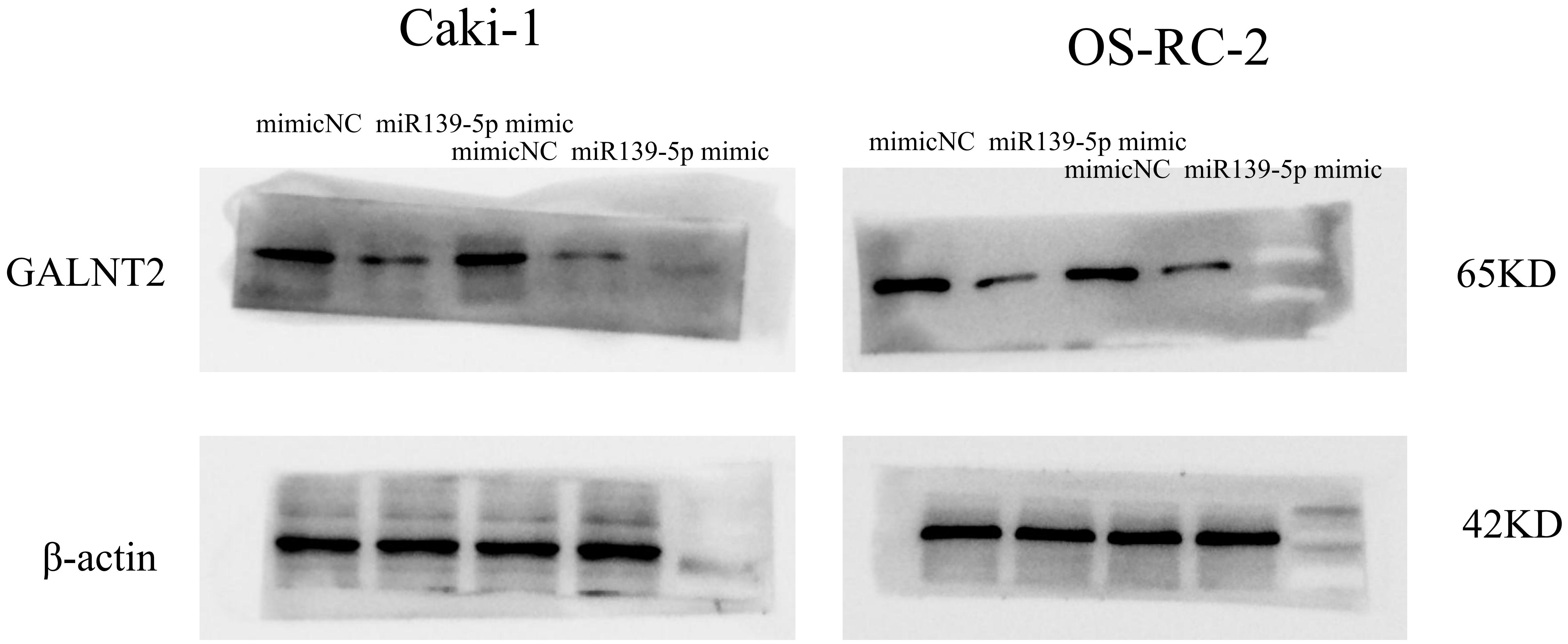
Caki-1
OS-RC-2
mimicNC miR139-5p mimic
mimicNC miR139-5p mimic
mimicNC miR139-5p mimic
mimicNC miR139-5p mimic
GALNT2
65KD
β-actin
42KD

GALNT2（wt）-psiCHECK-2

agatctgcgcagcaccatggcctgaaataacctctgaaagaggaacttggttaggtaccttctgaggcggaaagaaccagctgtggaatgtgt
gtcagttagggtgtggaaagtccccaggctccccagcaggcagaagtatgcaaagcatgcatctcaattagtcagcaaccaggtgtggaaag
tccccaggctccccagcaggcagaagtatgcaaagcatgcatctcaattagtcagcaaccatagtcccgcccctaactccgcccatcccgcc
cctaactccgcccagttccgcccattctccgccccatggctgactaattttttttatttatgcagaggccgaggccgcctcggcctctgagctattc
cagaagtagtgaggaggcttttttggaggcctaggcttttgcaaaaagcttgattcttctgacacaacagtctcgaacttaagctgcagaagttg
gtcgtgaggcactgggcaggtaagtatcaaggttacaagacaggtttaaggagaccaatagaaactgggcttgtcgagacagagaagactct
tgcgtttctgataggcacctattggtcttactgacatccactttgcctttctctccacaggtgtccactcccagttcaattacagctcttaaggctaga
gtacttaatacgactcactataggctagccaccatggcttccaaggtgtacgaccccgagcaacgcaaacgcatgatcactgggcctcagtgg
tgggctcgctgcaagcaaatgaacgtgctggactccttcatcaactactatgattccgagaagcacgccgagaacgccgtgatttttctgcatg
gtaacgctgcctccagctacctgtggaggcacgtcgtgcctcacatcgagcccgtggctagatgcatcatccctgatctgatcggaatgggta
agtccggcaagagcgggaatggctcatatcgcctcctggatcactacaagtacctcaccgcttggttcgagctgctgaaccttccaaagaaaa
tcatctttgtgggccacgactgggggggcttgtctggcctttcactactcctacgagcaccaagacaagatcaaggccatcgtccatgctgagag
tgtcgtggacgtgatcgagtcctgggacgagtggcctgacatcgaggaggatatcgccctgatcaagagcgaagagggcgagaaaatggt
gcttgagaataacttcttcgtcgagaccatgctcccaagcaagatcatgcggaaactggagcctgaggagttcgctgcctacctggagccatt
caaggagaagggcgaggttagacggcctaccctctcctggcctcgcgagatccctctcgttaagggaggcaagcccgacgtcgtccagatt
gtccgcaactacaacgcctaccttcgggccagcgacgatctgcctaagatgttcatcgagtccgaccctgggttcttttccaacgctattgtcga
gggagctaagaagttccctaacaccgagttcgtgaaggtgaagggcctccacttcagccaggaggacgctccagatgaaatgggtaagtac
atcaagagcttcgtggagcgcgtgctgaagaacgagcagtaattctaggcgatcgctcgagAGTCCCTCTCTCTGCTCCTC
CATTCGCAAGTGTCTTCCTGGGCCAGACTCCCCTCCACCTCATGTACTTGCTATATTGAG
GATGAAGTTTTCTATGGTGGGACACTAAATATAAAGCTATATAGAGAAAGAATGTACGG
TCAGTTCCCTATGGTTTCTGTAGATCATCGTCATCTTGTATATTCCCCACAAAGCCGTTC
GCAGCTTCCGGGAGAAGGGGCCAGAGCCCGGTGGGGCCAGTTTCTCACAGAGGGAG
GAGGTGGCCTCGTGCACTGTGGCCTTGGAGTTAGGAGCCACCCTGGGAGGTTGTGCC
GGTGCCATGCTCCTCCCTGTGTCTTGTATGAAAGGGGCCACTGTGTGTCTTCCTCCCCG
GCGGGAGCCCCACATGTAGGACAGCGGCCCCGAGGTGGAAGCCTGGCTGGAGGGCTG
CCCTATAGGTCTTCTCTTCCCGCCTCCCCTGCCATGCAACCAGATGTGTTGTGAGTGGG
CAGCGTGCCCCCAgcggccgctggccgcaataaaatatctttattttcattacatctgtgtgttggttttttgtgtgaggatctaaatga
gtcttcggacctcgcgggggccgcttaagcggtggttagggtttgtctgacgcggggggagggggaaggaacgaaacactctcattcggag
gcggctcggggtttggtcttggtggccacgggcacgcagaagagcgccgcgatcctcttaagcacccccccgccctccgtggaggcgggg
gtttggtcggcgggtggtaactggcgggccgctgactcgggcgggtcgcgcgccccagagtgtgaccttttcggtctgctcgcagaccccc
gggcggcgccgccgcggcggcgacgggctcgctgggtcctaggctccatggggaccgtatacgtggacaggctctggagcatccgcac
gactgcggtgatattaccggagaccttctgcgggacgagccgggtcacgcggctgacgcggagcgtccgttgggcgacaaacaccagga
cggggcacaggtacactatcttgtcacccggaggcgcgagggactgcaggagcttcagggagtggcgcagctgcttcatccccgtggccc
gttgctcgcgtttgctggcggtgtccccggaagaaatatatttgcatgtctttagttctatgatgacacaaaccccgcccagcgtcttgtcattggc
gaattcgaacacgcagatgcagtcggggcggcgcggtcccaggtccacttcgcatattaaggtgacgcgtgtggcctcgaacaccgagcg
accctgcagcgacccgcttaaaagcttggcattccggtactgttggtaaagccaccatggccgatgctaagaacattaagaagggccctgctc
ccttctaccctctggaggatggcaccgctggcgagcagctgcacaaggccatgaagaggtatgccctggtgcctggcaccattgccttcacc
gatgcccacattgaggtggacatcacctatgccgagtacttcgagatgtctgtgcgcctggccgaggccatgaagaggtacggcctgaacac
caaccaccgcatcgtggtgtgctctgagaactctctgcagttcttcatgccagtgctgggcgccctgttcatcggagtggccgtggcccctgct
aacgacatttacaacgagcgcgagctgctgaacagcatgggcatttctcagcctaccgtggtgttcgtgtctaagaagggcctgcagaagatc
ctgaacgtgcagaagaagctgcctatcatccagaagatcatcatcatggactctaagaccgactaccagggcttccagagcatgtacacattc
gtgacatctcatctgcctcctggcttcaacgagtacgacttcgtgccagagtctttcgacagggacaaaaccattgccctgatcatgaacagctc

tgggtctaccggcctgcctaagggcgtggccctgcctcatcgcaccgcctgtgtgcgcttctctcacgcccgcgaccctattttcggcaacca
gatcatccccgacaccgctattctgagcgtggtgccattccaccacggcttcggcatgttcaccaccctgggctacctgatttgcggctttcggg
tggtgctgatgtaccgcttcgaggaggagctgttcctgcgcagcctgcaagactacaaaattcagtctgccctgctggtgccaaccctgttcag
cttcttcgctaagagcaccctgatcgacaagtacgacctgtctaacctgcacgagattgcctctggcggcgccccactgtctaaggaggtggg
cgaagccgtggccaagcgctttcatctgccaggcatccgccagggctacggcctgaccgagacaaccagcgccattctgattaccccagag
ggcgacgacaagcctggcgccgtgggcaaggtggtgccattcttcgaggccaaggtggtggacctggacaccggcaagaccctgggagt
gaaccagcgcggcgagctgtgtgtgcgcggccctatgattatgtccggctacgtgaataaccctgaggccacaaacgccctgatcgacaag
gacggctggctgcactctggcgacattgcctactgggacgaggacgagcacttcttcatcgtggaccgcctgaagtctctgatcaagtacaag
ggctaccaggtggccccagccgagctggagtctatcctgctgcagcaccctaacattttcgacgccggagtggccggcctgcccgacgacg
atgccggcgagctgcctgccgccgtcgtcgtgctggaacacggcaagaccatgaccgagaaggagatcgtggactatgtggccagccag
gtgacaaccgccaagaagctgcgcggcggagtggtgttcgtggacgaggtgcccaagggcctgaccggcaagctggacgcccgcaaga
tccgcgagatcctgatcaaggctaagaaaggcggcaagatcgccgtgtaataattctagagtcggggcggccggccgcttcgagcagacat
gataagatacattgatgagtttggacaaaccacaactagaatgcagtgaaaaaaatgctttatttgtgaaatttgtgatgctattgctttatttgtaac
cattataagctgcaataaacaagttaacaacaacaattgcattcattttatgtttcaggttcagggggaggtgtgggaggttttttaaagcaagtaa
aacctctacaaatgtggtaaaatcgataaggatccaggtggcacttttcggggaaatgtgcgcggaacccctatttgtttatttttctaaatacattc
aaatatgtatccgctcatgagacaataaccctgataaatgcttcaataatattgaaaaaggaagagtatgagtattcaacatttccgtgtcgccctt
attcccttttttgcggcattttgccttcctgtttttgctcacccagaaacgctggtgaaagtaaaagatgctgaagatcagttgggtgcacgagtgg
gttacatcgaactggatctcaacagcggtaagatccttgagagttttcgccccgaagaacgttttccaatgatgagcacttttaaagttctgctatg
tggcgcggtattatcccgtattgacgccgggcaagagcaactcggtcgccgcatacactattctcagaatgacttggttgagtactcaccagtc
acagaaaagcatcttacggatggcatgacagtaagagaattatgcagtgctgccataaccatgagtgataacactgcggccaacttacttctga
caacgatcggaggaccgaaggagctaaccgcttttttgcacaacatgggggatcatgtaactcgccttgatcgttgggaaccggagctgaatg
aagccataccaaacgacgagcgtgacaccacgatgcctgtagcaatggcaacaacgttgcgcaaactattaactggcgaactacttactctag
cttcccggcaacaattaatagactggatggaggcggataaagttgcaggaccacttctgcgctcggcccttccggctggctggtttattgctgat
aaatctggagccggtgagcgtgggtctcgcggtatcattgcagcactggggccagatggtaagccctcccgtatcgtagttatctacacgacg
gggagtcaggcaactatggatgaacgaaatagacagatcgctgagataggtgcctcactgattaagcattggtaactgtcagaccaagtttact
catatatactttagattgatttaaaacttcatttttaatttaaaaggatctaggtgaagatcctttttgataatctcatgaccaaaatcccttaacgtgagt
tttcgttccactgagcgtcagaccccgtagaaaagatcaaaggatcttcttgagatcctttttttctgcgcgtaatctgctgcttgcaaacaaaaaa
accaccgctaccagcggtggtttgtttgccggatcaagagctaccaactctttttccgaaggtaactggcttcagcagagcgcagataccaaat
actgttcttctagtgtagccgtagttaggccaccacttcaagaactctgtagcaccgcctacatacctcgctctgctaatcctgttaccagtggctg
ctgccagtggcgataagtcgtgtcttaccgggttggactcaagacgatagttaccggataaggcgcagcggtcgggctgaacggggggttc
gtgcacacagcccagcttggagcgaacgacctacaccgaactgagatacctacagcgtgagctatgagaaagcgccacgcttcccgaagg
gagaaaggcggacaggtatccggtaagcggcagggtcggaacaggagagcgcacgagggagcttccagggggaaacgcctggtatcttt
atagtcctgtcgggtttcgccacctctgacttgagcgtcgatttttgtgatgctcgtcaggggggcggagcctatggaaaaacgccagcaacgc
ggcctttttacggttcctggccttttgctggccttttgctcacatggctcgac

GALNT2（mut）-psiCHECK-2

agatctgcgcagcaccatggcctgaaataacctctgaaagaggaacttggttaggtaccttctgaggcggaaagaaccagctgtggaatgtgt
gtcagttagggtgtggaaagtccccaggctccccagcaggcagaagtatgcaaagcatgcatctcaattagtcagcaaccaggtgtggaaag
tccccaggctccccagcaggcagaagtatgcaaagcatgcatctcaattagtcagcaaccatagtcccgcccctaactccgcccatcccgcc
cctaactccgcccagttccgcccattctccgccccatggctgactaattttttttatttatgcagaggccgaggccgcctcggcctctgagctattc
cagaagtagtgaggaggcttttttggaggcctaggcttttgcaaaaagcttgattcttctgacacaacagtctcgaacttaagctgcagaagttg
gtcgtgaggcactgggcaggtaagtatcaaggttacaagacaggtttaaggagaccaatagaaactgggcttgtcgagacagagaagactct
tgcgtttctgataggcacctattggtcttactgacatccactttgcctttctctccacaggtgtccactcccagttcaattacagctcttaaggctaga

gtacttaatacgactcactataggctagccaccatggcttccaaggtgtacgaccccgagcaacgcaaacgcatgatcactgggcctcagtgg
tgggctcgctgcaagcaaatgaacgtgctggactccttcatcaactactatgattccgagaagcacgccgagaacgccgtgatttttctgcatg
gtaacgctgcctccagctacctgtggaggcacgtcgtgcctcacatcgagcccgtggctagatgcatcatccctgatctgatcggaatgggta
agtccggcaagagcgggaatggctcatatcgcctcctggatcactacaagtacctcaccgcttggttcgagctgctgaaccttccaaagaaaa
tcatctttgtgggccacgactgggggggcttgtctggcctttcactactcctacgagcaccaagacaagatcaaggccatcgtccatgctgagag
tgtcgtggacgtgatcgagtcctgggacgagtggcctgacatcgaggaggatatcgccctgatcaagagcgaagagggcgagaaaatggt
gcttgagaataacttcttcgtcgagaccatgctcccaagcaagatcatgcggaaactggagcctgaggagttcgctgcctacctggagccatt
caaggagaagggcgaggttagacggcctaccctctcctggcctcgcgagatccctctcgttaagggaggcaagcccgacgtcgtccagatt
gtccgcaactacaacgcctaccttcgggccagcgacgatctgcctaagatgttcatcgagtccgaccctgggttcttttccaacgctattgtcga
gggagctaagaagttccctaacaccgagttcgtgaaggtgaagggcctccacttcagccaggaggacgctccagatgaaatgggtaagtac
atcaagagcttcgtggagcgcgtgctgaagaacgagcagtaattctaggcgatcgctcgagAGTCCCTCTCTCTGCTCCTC
CATTCGCAAGTGTCTTCCTGGGCCAGACTCCCCTCCACCTCATGTACTTGCTATATTGAG
GATGAAGTTTTCTATGGTGGGACACTAAATATAAAGCTATATAGAGAAAGAATGTACGG
TCAGTTCCCTATGGTTTTTATGGATCATCGTCATCTTGTATATTCCCCACAAAGCCGTTCG
CAGCTTCCGGGAGAAGGGGCCAGAGCCCGGTGGGGCCAGTTTCTCACAGAGGGAGG
AGGTGGCCTCGGCCTTGGAGTTAGGAGCCACCCTGGGAGGTTGTGCCGGTGCCATGCT
CCTCCCTGTGTCTTGTATGAAAGGGGCCACTGTGTGTCTTCCTCCCCGGCGGGAGCCC
CACATGTATACGCCGCAGGACAGCGGCCCCGAGGTGGAAGCCTGGCTGGAGGGCTGC
CCTATAGGTCTTCTCTTCCCGCCTCCCCTGCCATGCAACCAGATGTGTTGTGAGTGGGC
AGCGTGCCCCCAgcggccgctggccgcaataaaatatctttattttcattacatctgtgtgttggttttttgtgtgaggatctaaatgagt
cttcggacctcgcgggggccgcttaagcggtggttagggtttgtctgacgcggggggagggggaaggaacgaaacactctcattcggagg
cggctcggggtttggtcttggtggccacgggcacgcagaagagcgccgcgatcctcttaagcaccccccgccctccgtggaggcggggg
tttggtcggcgggtggtaactggcgggccgctgactcgggcgggtcgcgcgccccagagtgtgaccttttcggtctgctcgcagaccccg
ggcggcgccgccgcggcggcgacgggctcgctgggtcctaggctccatggggaccgtatacgtggacaggctctggagcatccgcacga
ctgcggtgatattaccggagaccttctgcgggacgagccgggtcacgcggctgacgcggagcgtccgttgggcgacaaacaccaggacg
gggcacaggtacactatcttgtcacccggaggcgcgagggactgcaggagcttcagggagtggcgcagctgcttcatccccgtggcccgtt
gctcgcgtttgctggcggtgtccccggaagaaatatatttgcatgtctttagttctatgatgacacaaaccccgcccagcgtcttgtcattggcga
attcgaacacgcagatgcagtcggggcggcgcggtcccaggtccacttcgcatattaaggtgacgcgtgtggcctcgaacaccgagcgacc
ctgcagcgacccgcttaaaagcttggcattccggtactgttggtaaagccaccatggccgatgctaagaacattaagaagggccctgctccctt
ctaccctctggaggatggcaccgctggcgagcagctgcacaaggccatgaagaggtatgccctggtgcctggcaccattgccttcaccgat
gcccacattgaggtggacatcacctatgccgagtacttcgagatgtctgtgcgcctggccgaggccatgaagaggtacggcctgaacaccaa
ccaccgcatcgtggtgtgctctgagaactctctgcagttcttcatgccagtgctgggcgccctgttcatcggagtggccgtggcccctgctaac
gacatttacaacgagcgcgagctgctgaacagcatgggcatttctcagcctaccgtggtgttcgtgtctaagaagggcctgcagaagatcctg
aacgtgcagaagaagctgcctatcatccagaagatcatcatcatggactctaagaccgactaccagggcttccagagcatgtacacattcgtg
acatctcatctgcctcctggcttcaacgagtacgacttcgtgccagagtctttcgacagggacaaaaccattgccctgatcatgaacagctctgg
gtctaccggcctgcctaagggcgtggccctgcctcatcgcaccgcctgtgtgcgcttctctcacgcccgcgaccctattttcggcaaccagatc
atccccgacaccgctattctgagcgtggtgccattccaccacggcttcggcatgttcaccaccctgggctacctgatttgcggctttcgggtggt
gctgatgtaccgcttcgaggaggagctgttcctgcgcagcctgcaagactacaaaattcagtctgccctgctggtgccaaccctgttcagcttct
tcgctaagagcaccctgatcgacaagtacgacctgtctaacctgcacgagattgcctctggcggcgccccactgtctaaggaggtgggcgaa
gccgtggccaagcgctttcatctgccaggcatccgccagggctacggcctgaccgagacaaccagcgccattctgattaccccagagggcg
acgacaagcctggcgccgtgggcaaggtggtgccattcttcgaggccaaggtggtggacctggacaccggcaagaccctgggagtgaac
cagcgcggcgagctgtgtgtgcgcggccctatgattatgtccggctacgtgaataaccctgaggccacaaacgccctgatcgacaaggacg
gctggctgcactctggcgacattgcctactgggacgaggacgagcacttcttcatcgtggaccgcctgaagtctctgatcaagtacaagggct
accaggtggccccagccgagctggagtctatcctgctgcagcaccctaacattttcgacgccggagtggccggcctgcccgacgacgatgc

cggcgagctgcctgccgccgtcgtcgtgctggaacacggcaagaccatgaccgagaaggagatcgtggactatgtggccagccaggtga
caaccgccaagaagctgcgcggcggagtggtgttcgtggacgaggtgcccaagggcctgaccggcaagctggacgcccgcaagatccg
cgagatcctgatcaaggctaagaaaggcggcaagatcgccgtgtaataattctagagtcggggcggccggccgcttcgagcagacatgata
agatacattgatgagtttggacaaaccacaactagaatgcagtgaaaaaaatgctttatttgtgaaatttgtgatgctattgctttatttgtaaccatt
ataagctgcaataaacaagttaacaacaacaattgcattcattttatgtttcaggttcagggggaggtgtgggaggttttttaaagcaagtaaaac
ctctacaaatgtggtaaaatcgataaggatccaggtggcacttttcggggaaatgtgcgcggaacccctatttgtttatttttctaaatacattcaaa
tatgtatccgctcatgagacaataaccctgataaatgcttcaataatattgaaaaaggaagagtatgagtattcaacatttccgtgtcgcccttattc
ccttttttgcggcattttgccttcctgtttttgctcacccagaaacgctggtgaaagtaaaagatgctgaagatcagttgggtgcacgagtgggtta
catcgaactggatctcaacagcggtaagatccttgagagttttcgccccgaagaacgttttccaatgatgagcacttttaaagttctgctatgtgg
cgcggtattatcccgtattgacgccgggcaagagcaactcggtcgccgcatacactattctcagaatgacttggttgagtactcaccagtcaca
gaaaagcatcttacggatggcatgacagtaagagaattatgcagtgctgccataaccatgagtgataacactgcggccaacttacttctgacaa
cgatcggaggaccgaaggagctaaccgcttttttgcacaacatgggggatcatgtaactcgccttgatcgttgggaaccggagctgaatgaa
gccataccaaacgacgagcgtgacaccacgatgcctgtagcaatggcaacaacgttgcgcaaactattaactggcgaactacttactctagct
tcccggcaacaattaatagactggatggaggcggataaagttgcaggaccacttctgcgctcggcccttccggctggctggtttattgctgata
aatctggagccggtgagcgtgggtctcgcggtatcattgcagcactggggccagatggtaagccctcccgtatcgtagttatctacacgacgg
ggagtcaggcaactatggatgaacgaaatagacagatcgctgagataggtgcctcactgattaagcattggtaactgtcagaccaagtttactc
atatatactttagattgatttaaaacttcatttttaatttaaaaggatctaggtgaagatcctttttgataatctcatgaccaaaatcccttaacgtgagtt
ttcgttccactgagcgtcagaccccgtagaaaagatcaaaggatcttcttgagatcctttttttctgcgcgtaatctgctgcttgcaaacaaaaaaa
ccaccgctaccagcggtggtttgtttgccggatcaagagctaccaactctttttccgaaggtaactggcttcagcagagcgcagataccaaata
ctgttcttctagtgtagccgtagttaggccaccacttcaagaactctgtagcaccgcctacatacctcgctctgctaatcctgttaccagtggctgc
tgccagtggcgataagtcgtgtcttaccgggttggactcaagacgatagttaccggataaggcgcagcggtcgggctgaacggggggttcgt
gcacacagcccagcttggagcgaacgacctacaccgaactgagatacctacagcgtgagctatgagaaagcgccacgcttcccgaaggga
gaaaggcggacaggtatccggtaagcggcagggtcggaacaggagagcgcacgagggagcttccagggggaaacgcctggtatctttat
agtcctgtcgggtttcgccacctctgacttgagcgtcgatttttgtgatgctcgtcaggggggcggagcctatggaaaaacgccagcaacgcg
gcctttttacggttcctggccttttgctggccttttgctcacatggctcgac
